# Demographics, psychiatric comorbidities, and hospital outcomes across eating disorder types in adolescents and youth: insights from US hospitals data

**DOI:** 10.3389/frcha.2024.1259038

**Published:** 2024-05-15

**Authors:** Sanobar Jaka, Sandesh Pokhrel, Archna Patel, Albulena Sejdiu, Sanjana Taneja, Sreshatha Vashist, Abimbola Arisoyin, Anil K. Bachu, Senthil Vel Rajan Rajaram Manoharan, Raja Mogallapu, Rikinkumar S. Patel

**Affiliations:** ^1^ Department of Population Health, School of Medicine, New York University, New York, NY, United States; ^2^ Department of Psychiatry, Nepal Medical College Teaching Hospital, Kathmandu, Nepal; ^3^ Department of Psychiatry, NIMS Medical College and Hospital, NIMS University, Jaipur, India; ^4^ Department of Psychiatry, Ss. Cyril and Methodius University, Skopje, North Macedonia; ^5^ Department of Psychiatry, Lady Hardinge Medical College and Associated Hospitals, Delhi, India; ^6^ Department of Psychiatry, N.C. Medical College and Hospital, Panipat, India; ^7^ Psychiatry Department, College of Medicine, University of Lagos, Lagos, Nigeria; ^8^ Department of Psychiatry, College of Medicine, University of Arkansas for Medical Sciences, Little Rock, AR, United States; ^9^ Department of Psychiatry and Behavioral Neurobiology, Heersink School of Medicine, University of Alabama at Birmingham, Birmingham, AL, United States; ^10^ Department of Behavioral Medicine and Psychiatry, School of Medicine, West Virginia University, Morgantown, WV, United States; ^11^ Department of Psychiatry & Behavioral Sciences, School of Medicine, Duke University, Durham, NC, United States

**Keywords:** eating and feeding disorders, anorexia nervosa, bulimia nervosa, avoidant/restrictive food intake disorder, adolescents, transitional-age youth

## Abstract

**Objective:**

The aim of our study was to delineate the differences in demographics, comorbidities, and hospital outcomes by eating disorder types in adolescents and transitional-age youth (15–26 years), and measure the association with psychiatric comorbidities.

**Methods:**

We conducted a cross-sectional study using the nationwide inpatient sample (2018–2019) and included 7,435 inpatients (age 12–24 years) with a primary diagnosis of eating disorders: anorexia nervosa (AN, 71.7%), bulimia nervosa (BN, 4.7%), avoidant/restrictive food intake disorder (ARFID, 9.5%), and other. We used independent logistic regression models controlled for demographics to evaluate the adjusted odds ratio association of comorbidities with eating disorder types.

**Results:**

The mean age of BN inpatients was 17.5 years, which was significantly higher compared to the total number of inpatients with eating disorders (15.9 years). Approximately four-fifths of the inpatients with AN and BN were female patients whereas ARFID was seen in a higher proportion of male patients (32.6% vs. 13.7% overall). Anxiety (57.5%) and depressive (47.3%) disorders were prevalent in the total number of inpatients with eating disorders, with suicidal behaviors seen significantly higher in BN (25.7% vs. 12.9% overall). The likelihood of obsessive compulsive–related disorder was higher in all eating disorder types, i.e., AN (OR 2.14), BN (OR 1.79), and ARFID (OR 1.74); however, anxiety (OR 1.52) and neurodevelopmental (OR 1.70) disorders were significantly higher in ARFID. In terms of hospital outcomes, inpatients with ARFID had a longer mean length of stay (13.7 days vs. 8.4 days in BN) and higher mean total charges ($87,747 vs. $44,882 in BN).

**Conclusion:**

Our findings identify notable demographic and clinical distinctions within inpatients diagnosed with AN, BN, and ARFID. Specifically, inpatients with BN belonging to older age brackets manifest elevated occurrences of depressive disorders and suicidal tendencies. ARFID is linked to prolonged hospitalization and increased costs, underscoring distinctive complexities in care. This highlights the significance of personalized interventions that account for demographic variations and psychiatric comorbidities, aiming to improve outcomes for diverse populations affected by eating disorders.

## Introduction

Approximately 30 million Americans are affected by eating disorders (EDs) at least once in their lifetime (1, 2), with an estimated economic burden of $64.7 billion per year (3). According to the Diagnostic and Statistical Manual of Mental Disorders, types of eating disorders include anorexia nervosa (AN), bulimia nervosa (BN), binge eating disorder (BED), avoidant restrictive food intake disorder (ARFID), and other specified feeding and EDs (4). In the United States, AN is a psychiatric disorder with one of the highest mortality rates, ranking second only to opioid overdose (4, 5). White females have the highest overall risk of developing eating disorders. However, Hispanic females are more likely than non-Hispanics to develop BN (6, 7). Similarly, black teenagers are more likely to exhibit binge eating and purging behaviors than white teenagers (6, 7). Profound and persistent disturbance in eating habits and associated disturbing thoughts and emotions are the hallmarks of eating disorders (4).

A systematic review encompassing studies conducted between 2000 and 2018 indicated that the worldwide lifetime prevalence of AN was up to 3.6% in females and up to 0.3% in males, while BN ranged up to 4.6% in females and up to 1.3% in males (8, 9). The prevalence of EDs in males has been underestimated, with recent research indicating a faster increase in rates compared to females. Male sex is associated with higher prevalence rates of extreme dieting and purging, while older age (over 45 years) is linked to increased rates of purging behaviors. Binge eating in males was associated with greater mental health-related quality of life impairment in 2008 compared to 1998, and with greater physical health-related quality of life impairment in regional areas compared to metropolitan areas. In addition, BED appears to be the most prevalent EDs in men, followed by BN and AN. Male adolescents tend to have lower prevalence rates of formal diagnostic criteria for EDs, with BED, BN, and AN being relatively low, suggesting a later onset of EDs in males compared to females, although some data suggest no differences in age of onset according to sex. Further research in diverse settings is needed to better understand ED prevalence and presentation in males (10–12). Furthermore, a systematic review of 30 observational studies conducted between 2013 and 2021 reported a worldwide prevalence range of 5%–22.5% for ARFID (13). Notably, individuals with EDs, particularly AN, exhibited significantly increased lifetime suicidality rates, with an 18-fold higher risk for AN and a seven-fold higher risk for BN (14, 15).

In a study investigating predictors of eating disorders, thin-ideal internalization, positive attitudes toward thinness, body dissatisfaction, dieting, overeating, and mental health treatment were all identified as factors predicting the development of BN, BED, and purging disorder (PD) (16). In addition, the most reliable evidence suggests that EDs are also associated with early traumatic and stressful experience (17). Furthermore, some studies indicate that differences in gonadal hormones contribute to disordered eating behavior in both sexes (18). In addition, theories proposing dysregulation of serotonin levels in the mesolimbic pathway and dopaminergic levels in the fronto-striatal circuit are widely recognized as contributors to the perception of body dysmorphia and subsequent symptoms of EDs (14). Therefore, EDs and the circumstances in which they arise are highly intricate and multifaceted.

EDs are a group of challenging mental illnesses themselves, and findings from several studies suggest approximately 55%–95% of patients diagnosed with eating disorders have a chance of developing comorbid psychiatric disorder (19), most commonly, depression, anxiety disorders, substance use disorders (SUD), and obsessive compulsive–related disorder (OCD) (20). Evidence suggests eating disorders are also associated with neurodevelopmental disorders and medical conditions, including cardiac, metabolic, gastrointestinal, and reproductive systems, causing difficulty in treatment and management (18, 19). It is imperative to identify comorbidities in eating disorders early to detect their potential impact on symptomatology and to find effective treatment courses.

The aim of the present retrospective cross-sectional study was to learn more about the differences between demographics, psychiatric comorbidities, medical comorbidities, and hospital outcomes associated with the spectrum of eating disorders in adolescents and transitional-age youth (TAY) (aged 15–26 years) and measure its association with psychiatric comorbidities in the United States.

## Methods

### Study sample

We conducted a cross-sectional study using the nationwide inpatient sample (NIS 2018–2019), and the data are obtained from more than 4,400 non-federal community hospitals from 48 states and the District of Columbia in the USA (21). The Clinical Classifications Software Refined (CCSR) was used to classify International Classification of Diseases, Tenth Revision (ICD-10)-coded diagnoses into clinical categories (22).

Our study included 7,435 inpatients (age 12–24 years) with a primary discharge diagnosis of eating and feeding disorders, and the sample was grouped based on the following: anorexia nervosa (ICD-10 codes: F50.00, F50.01, or F50.02), bulimia nervosa (ICD-10 code: F50.2), avoidant/restrictive food intake disorder (ICD-10 code: F50.82), and other eating disorders (ICD codes: F50.8, F50.89, or F50.9). The ICD-10 codes were confirmed as per AAPC, which is the nation's largest education and credentialing organization for medical coders, billers, auditors, and practice managers (22).

### Variables

The demographic variables included in the study were age, sex, race, and median household income. Comorbidities are co-diagnoses in the patient records, and we included both psychiatric and medical comorbidities. The psychiatric comorbidities that were extracted in our study using CCSR codes in parenthesis were anxiety disorders (MBD005), OCD (MBD006), neurodevelopmental disorders (MBD014), and suicidal behaviors including ideation/attempt/self-harm (MBD012). The medical comorbidities that were extracted in our study using CCSR codes in parenthesis were hypertension (CIR007 or CIR008), diabetes (END002), nutritional deficiencies (END007), cardiac dysrhythmias (CIR017), and fluid and electrolyte disorders (END011) (21). The CCSR categorizes International Classification of Diseases, 10th Revision, Clinical Modification/Procedure Coding System (ICD-10-CM/PCS) codes into clinically meaningful groups (21).

In the realm of ICD-10-CM diagnoses, the CCSR strikes a balance by preserving the clinical concepts found in the CCSR categories from ICD-9-CM while incorporating the precision offered by ICD-10-CM diagnoses through the creation of innovative clinical categories. All the included ICD-10-CM codes are shown in Supplementary Table 1.

The hospitalization outcomes of interest included the following: severity of illness that was measured using the all-patient refined diagnosis-related group (DRGs) (APR-DRGs) (21), length of stay (LOS), total charges, and disposition to skilled nursing/intermediate care facilities (SNF/ICF) (21).

### Statistical analysis

We compared the distributions of demographic characteristics, comorbidities, and hospital outcomes in inpatients by eating disorder type using descriptive statistics and linear-by-linear association tests for categorical variables, and ANOVA for continuous variables. Next, we used independent logistic regression models controlled for age, sex, race, and income to evaluate the adjusted odds ratio (aOR) association of psychiatric and medical comorbidities with eating disorder types. All analyses were conducted using Statistical Package for the Social Sciences (SPSS, IBM Corp., Armonk, NY, USA) and statistical significance was set at a two-sided *p*-value <0.05.

### Ethical approval

The NIS is a publicly available de-identified dataset from the Agency for Healthcare Research and Quality (AHRQ) (21). Therefore, as per the US Department of Health and Human Services, permission from the institutional review board was not required.

## Results

The majority of the study sample was formed by adolescents (aged 12–17 years, 75.5%), females (86.3%), and whites (71.4%), and those from high-income families with household incomes above the 50th percentile (73%). AN constituted about three-quarters of the total eating disorder inpatients followed by ARFID (9.5%) and BN (4.7%).

The mean age of inpatients with BN was 17.5 years, which was notably higher than the overall mean age of inpatients with eating disorders (15.9 years). A larger percentage of inpatients with BN fell within the 18–24-year age bracket (37.1% compared to 24.5% among all inpatients). Approximately four-fifths of the inpatients with AN and BN were females, whereas ARFID was seen in a higher proportion of males (32.6%) compared to the total number of inpatients with eating disorders (13.7%). Though the total eating disorder inpatients were majorly formed by the whites, the inpatients with BN were comparatively seen more in blacks (6% vs. 3.2% of total) and ARFID in the Hispanics (21.2% vs. 15.9%).

Anxiety disorders (57.5%) and depressive disorders (47.3%) were more prevalent in total inpatients with eating disorders. Inpatients with BN had a higher prevalence of depressive disorders (70%) and anxiety disorders (60.0%) compared to other eating disorders. In addition, suicidal behaviors, including ideations and attempt, were seen in 12.9% of total inpatients whereas there was a significantly higher prevalence seen in inpatients with BN (25.7%). Comorbid neurodevelopmental disorders were seen in 10.3% of total inpatients with eating disorders and it was significantly higher in ARFID (24.1%).

The prevalence of diabetes and hypertension in total inpatients with eating disorders was less than 1%. There was no significant difference in the prevalence of nutritional deficiencies and fluid and electrolyte disorders across the types of eating disorders. In addition, inpatients with AN had higher rates of cardiac dysrhythmias (4.2%) compared to those with ARFID and BN (1.4% each).

In terms of hospital outcomes, inpatients with ARFID had a longer mean LOS (13.7 days) and higher mean total charges ($87,747) compared to other eating disorders. Further, inpatients with BN had a shorter mean LOS (8.4 days) and lower mean total charges ($44,882), as shown in Table 1.

**Table 1:** Differences in demographics, comorbidities, and outcomes in inpatients hospitalized for eating disorders.

Variable	Total	Eating disorders				*P*-value
		Others	AN	BN	ARFID	
Number of inpatients	7,435	1,050	5,330	350	705	—
Mean age (SD)	15.9 (2.8)	15.9 (2.6)	15.9 (2.8)	17.5 (3.0)	15.8 (2.9)	<0.001
Age (%)						
12–17 years	75.5	81.4	74.9	62.9	78.0	<0.001
18–24 years	24.5	18.6	25.1	37.1	22.0	
Sex (%)						
Male	13.7	16.2	10.6	14.3	32.6	<0.001
Female	86.3	83.8	89.4	85.7	67.4	
Race (%)						
White	71.4	73.8	72.2	67.2	65.0	<0.001
Black	3.2	6.9	2.3	6.0	2.9	
Hispanic	15.9	12.4	15.9	16.4	21.2	
Other	9.4	6.9	9.7	10.4	10.9	
Median household income (%)						
Below 50th percentile	27.0	25.4	27.3	25.4	28.8	0.205
Above 50th percentile	73.0	74.6	72.7	74.6	71.2	
Psychiatric comorbidities (%)						
Depressive disorders	47.3	48.1	47.6	70.0	33.3	<0.001
Anxiety disorders	57.5	55.2	56.9	60.0	63.8	<0.001
Obsessive compulsive–related disorders	11.0	6.2	12.0	11.4	10.6	0.021
Neurodevelopmental disorders	10.3	14.8	7.4	12.9	24.1	<0.001
Suicidal behaviors	12.9	11.9	13.4	25.7	4.3	<0.001
Medical comorbidities (%)						
Hypertension	1.1	1.9	0.6	2.9	3.5	<0.001
Diabetes	0.3	1.0	0.2	1.4	0	0.048
Nutritional deficiencies	10.0	9.0	10.0	8.6	11.3	0.207
Cardiac dysrhythmias	4.5	9.0	4.2	1.4	1.4	<0.001
Fluid and electrolyte disorders	31.6	30.5	31.2	38.6	32.6	0.091
Hospital outcomes						
Mean LOS, in days (SD)	11.9 (12.9)	8.9 (7.1)	12.5 (13.6)	8.4 (8.3)	13.7 (14.7)	<0.001
Mean charges ($) (SD)	73,391 (80,085)	56,380 (48,196)	76,705 (78,921)	44,882 (44,938)	87,747 (1,22,318)	<0.001
Major loss of body functioning	59.9	37.6	67.8	41.4	41.8	0.001
Discharge to SNF/ICF	12.3	10.5	13.6	10.0	6.4	<0.001

Among the psychiatric comorbidities, inpatients with BN had the highest likelihood of depressive disorders (OR 2.31) and suicidal behaviors (OR 2.53) compared to other types of eating disorders. When the likelihood of anxiety disorders was significantly higher in ARFID (OR 1.52) then OCD was much higher in those with AN (OR 2.14) followed by BN (OR 1.79) and ARFID (OR 1.74). Lastly, of all types of eating disorders, those with ARFID had a higher association with comorbid neurodevelopmental disorders (OR 1.70).

In terms of medical comorbidities, all types of eating disorders had a lower association with cardiac dysrhythmias, whereas the risk of hypertension was significantly higher in ARFID (OR 2.15). Diabetes, nutritional deficiencies, and fluid and electrolyte disorders had no significant association with types of eating disorders, as shown in Table 2.

**Table 2:** Odds of association with comorbidities by eating disorders.

Variable	Adjusted odds ratio (95% confidence interval)		
	AN	BN	ARFID
Psychiatric comorbidities			
Depressive disorders	0.92 (0.80–1.06)	2.31 (1.77–3.02)	0.51 (0.41–0.62)
Anxiety disorders	0.97 (0.84–1.12)	1.07 (0.83–1.38)	1.52 (1.24–1.86)
Obsessive compulsive–related disorders	2.14 (1.62–2.82)	1.79 (1.15–2.78)	1.74 (1.21–2.50)
Neurodevelopmental disorders	0.52 (0.42–0.64)	0.89 (0.61–1.31)	1.70 (1.31–2.21)
Suicidal behaviors	1.22 (0.98–1.51)	2.53 (1.85–3.48)	0.38 (0.25–0.58)
Medical comorbidities			
Hypertension	0.28 (0.15–0.52)	1.38 (0.63–3.04)	2.15 (1.15–4.03)
Diabetes	0.20 (0.08–0.49)	1.39 (0.46–4.27)	<0.001
Nutritional deficiencies	1.19 (0.94–1.53)	1.11 (0.72–1.73)	1.38 (0.99–1.91)
Cardiac dysrhythmias	0.44 (0.34–0.57)	0.14 (0.06–0.35)	0.17 (0.09–0.33)
Fluid and electrolyte disorders	1.01 (0.87–1.18)	1.17 (0.89–1.53)	1.09 (0.88–1.35)

## Discussion

Our research revealed that nearly 80% of patients hospitalized primarily for AN and BN were females. ARFID was more prevalent in males. White ethnicity comprised the majority of total inpatients with eating disorders, but BN was relatively more common among black ethnicity inpatients and ARFID was higher among Hispanic inpatients. Anxiety and depressive disorders were the most prevalent psychiatric comorbidities in the overall inpatient sample with eating disorders, and suicidal behaviors were more common in BN compared to other types of eating disorders. Among medical comorbidities, electrolyte disorders and nutritional deficiencies were prevalent, while hypertension, diabetes, and cardiac dysrhythmias had very low prevalence.

Coinciding with our findings, 5.5%–17.9% of young women and 0.6%–2.4% of young men experience Diagnostic and Statistical Manual of Mental Disorders (DSM-5) eating disorders by early adulthood (23). Lifetime rates of DSM-5 anorexia nervosa, bulimia nervosa, binge eating disorder, and other specified or unspecified feeding/eating disorders vary among genders, with females generally experiencing higher rates, as also seen with our findings. According to a nationwide survey in the US, individuals who sought emergency room care primarily for eating disorders were more frequently young females and originated from higher-income households (24). A recent study by Dinkler and Bryant-Waugh provided detailed insights into the prevalence of ARFID across age groups, with rates in the range of 0.3%–15.5% in children and adolescents, and 0.8%–4.5% among adults (25).

A retrospective study examining charts revealed that patients with ARFID were younger (12.9 years vs. 15.6 and 16.5 years for AN and BN), had a longer duration of illness (33.3 months vs. 14.5 and 23.5 months), and were more likely to be male (29% vs. 15% and 6%) compared to patients with AN and BN (26). Our study yielded similar findings, with comparable mean ages between inpatients with ARFID and AN, while the mean age of inpatients with BN was slightly higher.

Our study revealed that white ethnicity was predominant among total inpatients with eating disorders. Among ethnicities, BN was relatively more prevalent among black individuals, while ARFID was more commonly observed in Hispanic individuals. In addition, findings from the largest national sample of American adults indicated that non-Hispanic black and Hispanic respondents had a significantly lower likelihood of being diagnosed with AN compared to white respondents (27). Nevertheless, there is a lack of studies examining variations in ethnicity within eating disorders. In a recent review spanning two decades of studies about eating disorders, findings indicated that white participants constituted approximately 70% of samples providing race and ethnicity data, with Hispanic participants making up approximately 10%. Individuals from all other racial and ethnic backgrounds each comprised less than 5% of the samples (28).

Eating disorders often coexist with other psychiatric conditions (29). However, prevalence estimates exhibit significant variation due to methodological differences across studies, including variations in population, diagnostic methods, and sampling procedures. Anxiety disorders are the most prevalent psychiatric comorbidity, affecting those with EDs (19). Anxiety disorders often precede the onset of eating disorders, particularly prevalent in women with binge eating disorder and in men with BN (19, 29). Mood disorders, such as depression, are also highly comorbid, affecting up to 54% of those with EDs (19). Approximately 32%–39% of individuals with AN and 36%–50% with BN also receive a diagnosis of comorbid depressive disorders (30, 31). Observations suggest that individuals with ARFID are more likely to have a co-diagnosis of anxiety disorder but less likely to have a mood disorder compared to individuals with AN or BN (26, 32). Consistent with this literature, our study found that anxiety disorders were more prevalent, followed by depressive disorders, in total inpatients with EDs. The likelihood of anxiety disorders was significantly higher in individuals with ARFID (by 1.5 times), and OCD was much higher in those with AN (by two times), followed by BN and ARFID. Shared genetic and neurobiological factors link EDs and OCD, suggesting common genetic features (33, 34). Neurobiological processes, like basal ganglia activation and serotonin–glutamate interactions, are implicated in both disorders (35). Anxiety and fear play significant roles in both, involving fear learning and safety behaviors and the amygdala are involved in fear responses in both conditions (36). However, research primarily focuses on AN and OCD, leaving other EDs and OCD subtypes understudied and is crucial to understand shared mechanisms across different EDs and OCD subtypes and to accurately classify OCD (33).

The present study found that 12.9% of all inpatients with eating disorders exhibited suicidal behaviors, with a notably higher occurrence among those diagnosed with BN. Patel et al. reported a 3–5 times increase in lifetime suicidality rates in adolescents with eating disorders (14), while Swanson et al. found a higher prevalence of lifetime suicidal attempts with BN (35.1%) compared to those with AN (20.6%) (37). In addition, our findings indicated that inpatients diagnosed with BN had double the likelihood of depressive and suicidal behaviors compared to other categories of eating disorders. Moreover, the prevalence of concurrent neurodevelopmental disorders was higher among inpatients with ARFID, and a significant association between both conditions was strengthened by 1.7 times. These results align with previous research highlighting an elevated presence of neurodevelopmental disorders in individuals with ARFID (38, 39). Individuals with ARFID often have comorbid conditions like autism spectrum disorder (ASD), resembling patterns seen in other feeding disorders. ASD-related characteristics, such as food selectivity due to sensory sensitivity, are common in ARFID. This suggests a potential link between ASD and restrictive EDs like ARFID and AN (40).

The occurrence of diabetes and hypertension among inpatients with eating disorders was below 1%. It is not uncommon for individuals with both type 1 and type 2 diabetes to develop eating disorders, with those with type 1 diabetes being more prone to complications (41). Similarly, individuals with type 2 diabetes can also develop eating disorders, and BED is the most frequently diagnosed condition in this group (41). Our study reported that inpatients with AN had higher cardiac dysrhythmias rates than those with ARFID and BN. According to Mehler and Brown, bradycardia and hypotension are among the most common physical findings in patients with AN, with bradycardia seen in up to 95% of patients (42). Regarding hospital outcomes, inpatients with ARFID had a longer mean LOS, which is similar to the study's finding that those with ARFID had a longer hospitalization stay, thought to be due to increased reliance on enteral feeding and lower starting calorie goals early in the admission (43).

The sample analyzed in this study represents the NIS population of non-federal community hospitals in the US, potentially introducing a selection bias and reflecting a higher severity of symptoms (21). Consequently, it may not accurately mirror the broader population, where outpatient settings commonly address mild to moderate symptoms in patients with eating disorders. Furthermore, being a cross-sectional study, it may not thoroughly explore confounding and causal factors. Lastly, the quality of clinical assessment and diagnostic accuracy cannot be regulated due to the administrative nature of the data, and the transformation from ICD-10 diagnostic codes to DSM categories poses a potential source of bias.

Nonetheless, the study benefits from a large sample size drawn from a nationwide inpatient hospital dataset of the US, providing highly valuable information about comorbidities in patients with eating disorders with severe symptomatology. The use of logistic regression allowed for a more comprehensive analysis of the dataset. Another distinctive aspect of this study is that it comprehensively examines the entire spectrum of eating disorders, encompassing anorexia, bulimia, and ARFID, in the nationwide sample of inpatients. The study specifically investigated variables such as length of stay, which can aid in developing effective care plans for managing patients with eating disorders.

The results of this study were showcased in poster format at the American Child and Adolescent Psychiatry Conference in October 2023. In addition, an abstract summarizing the study was published in the *American Child and Adolescent Psychiatry Journal* (44).

## Conclusion

Our findings identify notable demographic and clinical distinctions within inpatients diagnosed with AN, BN, and ARFID. Specifically, inpatients with BN belonging to older age brackets manifest elevated occurrences of depressive disorders and suicidal tendencies. ARFID is linked to prolonged hospitalization and increased costs, underscoring distinctive complexities in care. This highlights the significance of personalized interventions that account for demographic variations and psychiatric comorbidities, aiming to improve outcomes for diverse populations affected by eating disorders.

## Data Availability

The raw data supporting the conclusions of this article will be made available by the authors, without undue reservation.
