# Supplementary material for: Demographics, psychiatric comorbidities, and hospital outcomes across eating disorder types in adolescents and youth: insights from US hospitals data
Source: Front Child Adolesc Psychiatry. 2024 May 15;3:1259038. doi: 10.3389/frcha.2024.1259038 (PMC11732058; doi:10.3389/frcha.2024.1259038)
Supplement: Supplementary file 1 [file Table1.docx]

| Diagnoses | ICD 10 Codes |
| --- | --- |
| Depressive disorders | F0632,F320, F321, F328, F3289, F329, F330, F331, F3341, F338, F339 |
| Anxiety disorders | F064, F4000, F4001, F4002, F4010, F4011, F40210, F40218, F40220, F40228, F40230  F40231, F40232, F40233, F40240, F40241  F40242, F40243, F40248, F40290, F40291  F40298, F408, F409, F410, F411, F413, F418  F419, F930, F940 |
| Obsessive compulsive -related disorders | F42, F422, F423, F424, F428, F429, F4522  F633 |
| Neurodevelopmental disorders | F70, F71, F72, F73, F78, F79, F800, F801  F802, F8081, F8082, F8089, F809, F810, F812  F8181, F8189, F82, F840, F842, F843, F845  F848, F849, F88, F89, F900, F901, F902, F908, F909, F984, F985, F988 |
| Suicidal behaviors | R45851, T1491, T1491XA, T1491XD,  T360X2A, T361X2A, T363X2A, T364X2A,  T366X2A, T367X2A, T368X2A, T3692XA,  T370X2A, T371X2A, T372X2A, T373X2A,  T374X2A, T375X2A, T3792XA, T380X2A,  T381X2A, T382X2A, T383X2A, T384X2A,  T385X2A, T386X2A, T387X2A, T38802A,  T38812A, T38892A, T38992A, T39012A,  T39092A, T391X2A, T392X2A, T39312A,  T39392A, T394X2A, T398X2A, T3992XA,  T400X2A, T401X2A, T402X2A, T403X2A,  T404X2A, T40602A, T40692A, T407X2A,  T408X2A, T40992A, T410X2A |
| Hypertension | I10, I150, I151, I152, I158, I159, I160, I161, I169, |
| Diabetes | E099, E109, E119, E139 |
| Nutritional deficiencies | E500  E502  E503  E504  E505  E506  E507  E508  E509  E5111  E5112  E512  E518  E519  E52  E530  E531  E538  E539  E54  E550  E559  E560  E561  E568  E569  E58  E59  E60  E610  E611  E612  E613  E614  E615  E616  E617  E618  E619  E630  E631  E638  E639 |
| Cardiac dysrhythmias | I470  I471  I472  I479  I480  I481  I4811  I4819  I482  I4820  I4821  I483  I484  I4891  I4892  I491  I492  I493  I4940  I4949  I495  I498  I499 |
| Fluid and electrolyte disorders | E860  E861  E869  E870  E871  E872  E873  E874  E875  E876  E8770  E8771  E8779  E878 |
